# Theory on Extremal Nucleon Heuristics to Psychiatry

**DOI:** 10.1192/j.eurpsy.2023.637

**Published:** 2023-07-19

**Authors:** Y. Pachankis

**Affiliations:** Independent, Chongqing, China

## Abstract

**Introduction:**

The theorization focuses on the climate change’s influence to neurobiology. In modern societies, environmental nucleon generates in everyday activities from computers to industrial pollution. The subtle psychiatric changes can be categorized into: 1) the change of media in consciousness formation processes from cognition, such as from paper to electronic reading and from linguistics to coding; 2) activity changes in local reciprocal environment especially in places undergoing industrialization or developmental energy sources; 3) global exchanges underlying the current definitions of climate change but also taken into consideration of media change in cognitive behaviors; and 4) changes from outer space environment to the effects of global-to-local changes.

**Objectives:**

The objective of the theorization seeks to develop a heuristic paradigm to quantify the climate change’s effects to psychiatry from a neurobiological perspective. Albeit climate change is a complex topical issue, especially regarding the multivariable sources and traditional paradigms of case studies in the psychological and medical sciences, common sources of impacts to psychiatric public health in collective behaviors have been less of a focus. With the higher order of autonomous human functioning governed by the brains, the theorization in psychiatric public health hopes to quantify environmental impacts to brain functionings.

**Methods:**

The theorization accumulated from nonproliferation research and the researcher’s developments in dopamine treatments in the high risk social-natural environment on depression. Inspired by the explicit review on electronic warfare’s impact on public health and astronomical research with proton decay outcome, a correlative theorization emerged between the cosmic decay and biodiversity in biochemistry. The theorization draws on developmental psychology to the nucleon heuristics in data research in cosmology, with prior experience documenting proliferation by applied quantum chromodynamics. Psychiatric data can be retrieved from relevant clinical settings of equivalent multi-wavelength brain scans as samples.

**Results:**

From the perspective of cosmology, extremal graph theory can sample climate change on earth’s plasma from cosmic changes. This means earth’s dipole shifts to quantitative local population can be plotted, similar to the wild life researches in birds’ migration pattern changes. Local variants mainly derive from energy source types and energy consumption, however, *bona fide* data can hardly be retrieved due to deliberate transgressions for certain dire areas. Normative research can be conducted with cross-disciplinary collaborations with due consideration to privacy in public health research ethics.

**Conclusions:**

Environmental monitoring and psychiatric effects in developmental psychology are necessary in fundamental research on human security. This would increase some certainties and predictabilities for human development.

**Disclosure of Interest:**

None Declared

